# FOXM1-AKT Positive Regulation Loop Provides Venetoclax Resistance in AML

**DOI:** 10.3389/fonc.2021.696532

**Published:** 2021-07-26

**Authors:** Mikhail S Chesnokov, Soheila Borhani, Marianna Halasi, Zarema Arbieva, Irum Khan, Andrei L. Gartel

**Affiliations:** ^1^ Department of Medicine, University of Illinois at Chicago, Chicago, IL, United States; ^2^ Department of Surgery, Massachusetts General Hospital, Boston, MA, United States; ^3^ Genome Research Core, University of Illinois at Chicago, Chicago, IL, United States

**Keywords:** FOXM1, AKT pathway, acute myeloid leukemia, positive feedback loop, drug resistance, *HOXA* gene family, venetoclax

## Abstract

Forkhead box protein M1 (FOXM1) is a crucial regulator of cancer development and chemoresistance. It is often overexpressed in acute myeloid leukemia (AML) and is associated with poor survival and reduced efficacy of cytarabine therapy. Molecular mechanisms underlying high FOXM1 expression levels in malignant cells are still unclear. Here we demonstrate that AKT and FOXM1 constitute a positive autoregulatory loop in AML cells that sustains high activity of both pro-oncogenic regulators. Inactivation of either AKT or FOXM1 signaling results in disruption of whole loop, coordinated suppression of FOXM1 or AKT, respectively, and similar transcriptomic changes. AML cells with inhibited AKT activity or stable FOXM1 knockdown display increase in *HOXA* genes expression and *BCL2L1* suppression that are associated with prominent sensitization to treatment with Bcl-2 inhibitor venetoclax. Taken together, our data indicate that AKT and FOXM1 in AML cells should not be evaluated as single independent regulators but as two parts of a common FOXM1-AKT positive feedback circuit. We also report for the first time that FOXM1 inactivation can overcome AML venetoclax resistance. Thus, targeting FOXM1-AKT loop may open new possibilities in overcoming AML drug resistance and improving outcomes for AML patients.

## Introduction

Forkhead box protein M1 (FOXM1) has emerged as a key contributor to cell proliferation and renewal as well as tumorigenesis. FOXM1 overexpression occurs in many human cancers, including hematopoietic malignancies, and plays a crucial role in cancer development and progression, serving both as a potent effector of tumor development and prognostic indicator for patients (1–3). High FOXM1 levels are generally associated with chemoresistance of malignant cells, aggressive tumor phenotype, and poor prognosis due to decreased efficacy of common therapeutic strategies (2, 3).

Acute myeloid leukemia (AML) accounts for the majority of all acute leukemia cases in adults and manifests significant clinical and biological heterogeneity. High FOXM1 expression in AML cells is directly associated with treatment resistance and inferior survival in AML patients (4, 5). Our discovery of interaction between FOXM1 and nucleophosmin (NPM, encoded by *NPM1* gene) suggested that high chemosensitivity of AML cells expressing mutant NPM (NPM^mut^) may be closely associated with FOXM1 inactivation due to its relocalization to cytoplasm (4–6). Thus, FOXM1 is a promising target in AML, and its inactivation may potentially be used to overcome drug resistance (7).

The molecular mechanisms contributing to high FOXM1 levels in cancer, including AML, are still not completely understood. The data accumulated from a variety of solid tumors demonstrated that FOXM1 expression is substantially controlled by phosphoinositide-3-kinase (PI3K)-AKT-Forkhead box protein O3 (FOXO3) pathway. AKT is a well-known oncogenic kinase that phosphorylates and inactivates FOXO3 protein, thus promoting FOXM1 expression (8). High activity of AKT pathway is observed in most AML cases and is associated with chemoresistance and adverse prognosis. AKT activation is often caused by activating mutations or amplifications of upstream AKT regulators (with fms like tyrosine kinase 3 internal tandem duplications (FLT3-ITD) being most common), but it can occur in the absence of clear driver events (9). A growing body of evidence suggests that FOXM1 may reciprocally promote AKT activation, thus establishing a positive feedback loop (1). Such loop could potentially sustain elevated activity of both regulators and ultimately promote AML chemoresistance. However, the existence of FOXM1-AKT positive feedback circuit was never confirmed in AML.

In the present study we report a positive autoregulatory loop between FOXM1 and AKT in human AML cell lines and demonstrate that inhibition of either of the two regulators exerts similar effects upon gene expression patterns. We also report for the first time that FOXM1 inactivation in AML cells can sensitize them to Bcl-2 inhibitor venetoclax that is considered as a very promising agent for AML treatment.

## Materials and Methods

### Cell Cultures

KG-1 human AML cell line was purchased from American Type Culture Collection (ATCC, Manassas, VA, USA). THP-1 human AML cell line was provided by Dr. Nadim Mahmud (University of Illinois at Chicago, Chicago, IL, USA). KG-1 cells were cultured in IMDM medium (Thermo Fisher Scientific, Waltham, MA, USA) supplemented with 10% fetal bovine serum (Thermo Fisher Scientific), 100 U/mL penicillin (Lonza, Basel, Switzerland), and 100 μg/mL streptomycin (Lonza). THP-1 cells were cultured in RPMI-1640 medium supplemented with 50 μM β-mercaptoethanol (Bio-Rad, Hercules, CA, USA), 10% fetal bovine serum (Thermo Fisher Scientific), 100 U/mL penicillin (Lonza), and 100 μg/mL streptomycin (Lonza). All cell lines were confirmed to be mycoplasma-negative by routine testing using PCR detection. Cells were subcultured upon reaching the concentration of 1 000 000 cells/mL of growth medium.

### Chemical Compounds and Drugs

MK-2206 (APExBIO Technology, Houston, TX, USA), LY294002 (APExBIO Technology), RCM-1 (R&D Systems, Minneapolis, MN, USA), and venetoclax (Selleck Chemicals, Houston, TX, USA) were dissolved in DMSO. Puromycin (LKT Laboratories, St Paul, MN, USA) was dissolved in water.

### Drug Treatment of Cultured Cells

Cells were harvested by centrifugation at 200 g for 5 minutes and viable cells were counted in presence of 0.4% Trypan Blue (Thermo Fisher Scientific). Cell treatment was performed by resuspending 2 000 000 viable cells in growth medium containing selected concentrations of drugs. Control samples were treated with vehicle only, vehicle concentration did not exceed 0.3%. After incubation for the desired periods of time, the cells were immediately harvested by centrifugation at 200 g for 5 minutes, washed once with cold phosphate-buffered saline (PBS), pelleted by centrifugation at 200 g for 5 minutes, and processed as described below.

### FOXM1 Expression Knockdown in KG-1 Cells

KG-1 cells were harvested by centrifugation, washed once with Hank’s balanced salt solution and resuspended in fresh IMDM medium supplemented with 10% fetal bovine serum (Thermo Fisher Scientific). Cells were seeded into 12-well tissue culture plates and incubated with MISSION lentiviral particles carrying pLKO.1 vector encoding control non-target shRNA or shRNA against human FOXM1 transcripts (MilliporeSigma, Burlington, MA, USA) in presence of 10 mg/mL polybrene (MilliporeSigma) for manufacturer. Transduced cells were selected by cultivation in presence of puromycin for 7 days and then cultured as described above. These stable FOXM1-deficient KG-1 cells were previously described and characterized (5).

### Protein Immunoblotting

Total protein samples were purified from cells using Pierce RIPA lysis buffer (Thermo Fisher Scientific) supplemented with Halt protease inhibitor cocktail (Thermo Fisher Scientific), 2 mM sodium orthovanadate (New England Biolabs Inc., Ipswich, MA, USA), and 5 mM sodium fluoride (MilliporeSigma) according to manufacturer’s protocol. Protein concentrations were estimated using Bio-Rad Protein Assay (Bio-Rad). 15-30 μg of total protein were mixed with Laemmli sample buffer (Bio-Rad) containing β-mercaptoethanol (Bio-Rad, final concentration 2.5%), heated at 98°C for 10 minutes, and separated in hand-cast 12% SDS-polyacrylamide gels. After the electrophoretic separation, the proteins were transferred to Immobilon-Psq PVDF membrane (MilliporeSigma). Membranes were washed with tris-buffered saline (TBS, Alfa Aesar, Haverhill, MA, USA) for 10 minutes, blocked with 5% bovine serum albumin (BSA, MilliporeSigma) in TBS with 0.1% Tween-20 (TBST, Thermo Fisher Scientific), and probed with the primary antibodies diluted in 5% BSA in TBST overnight at 4°C (see **Supplementary Table 1** for the list of antibodies used). Membranes were washed with TBST 3 times, 10 minutes each, and probed with HRP-conjugated secondary antibodies diluted in 5% skim milk (Research Products International, Mt Prospect, IL, USA) in TBST for 1 hour at room temperature. Membranes were washed with TBST 3 times, 10 minutes each, protein bands were developed using SuperSignal West Pico PLUS substrate (Thermo Fisher Scientific) and detected using ChemiDoc MP System (Bio-Rad). Protein band quantitation was performed using ImageLab software package (Bio-Rad).

### RT-qPCR Analysis of Gene Expression

Total RNA was isolated from cells using TRIzol reagent (Thermo Fisher Scientific) and the PureLink RNA Mini Kit (Thermo Fisher Scientific) with additional on-column DNAse treatment according to manufacturer’s instructions. RNA samples were quantified using NanoDrop One Spectrophotometer (Thermo Fisher Scientific). Reverse transcription was performed using High-Capacity cDNA Reverse Transcription Kit with RNase Inhibitor (Thermo Fisher Scientific), 500 ng of total RNA were used per reaction. Quantitative PCR analysis of gene expression levels was performed in ViiA 7 Real-Time PCR System (Thermo Fisher Scientific) using PowerUp SYBR Green Master Mix (Thermo Fisher Scientific) and primers listed in **Supplementary Table 2**. Amplification was performed according to manufacturer’s Fast Mode recommendations for 35 cycles, reaction specificity was checked by melt curve analysis and agarose electrophoresis. Reaction efficiency was evaluated using standard curve approach and was within 95-105% for all primers. Transcript abundance was estimated using Pfaffl’s method (10), *GAPDH* was used as reference gene for normalization.

### Full Transcriptome RNA-Seq

RNA samples were analyzed for integrity using Agilent 4200 TapeStation (Agilent Technologies, Santa Clara, CA, USA). Levels of remaining DNA were checked using Qubit fluorometer (Thermo Fisher Scientific). DNA amounts did not exceed 10% of the total amount of nucleic acid.

Sequencing libraries for Illumina sequencing were prepared in one batch in 96-well plate using Stranded CORALL total RNAseq library prep kit with RiboCop HMR rRNA Depletion Kit (Lexogen, Vienna, Austria). In brief, 260-660 nanograms of total RNA were used for the first rRNA depletion step, then followed by library generation initiated with random oligonucleotide primer hybridization and reverse transcription. No prior RNA fragmentation was done, as the insert size was determined by proprietary size restricting method. Next, the 3’ ends of first-strand cDNA fragments were ligated with a linker containing Illumina-compatible P5 sequences and Unique Molecular Identifiers. During the following steps of second strand cDNA synthesis and dual-strand cDNA amplification, i7 and i5 indices as well as complete adapter sequences required for cluster generation were added. A number of PCR amplification cycles was 12, as determined by qPCR using a small pre-amplification library aliquot for each individual sample.

Final amplified libraries were purified, quantified, and average fragment sizes confirmed to be 330 bp by gel electrophoresis using Agilent 4200 TapeStation (Agilent Technologies). Concentration of the final library pool was confirmed by qPCR, and then subjected to test sequencing in order to check sequencing efficiencies and adjust accordingly proportions of individual libraries. Sequencing was carried out on NovaSeq 6000, S4 flowcell (Illumina, San Diego, CA, USA), approximately 30 M 2x150 bp clusters per sample.

### Bioinformatical Analysis of RNA-Seq Data

Analysis of raw RNA-seq data was performed by Research Informatics Core at the University of Illinois at Chicago. Raw reads were aligned to the human hg38 reference genome in a splice-aware manner using the STAR aligner (11). ENSEMBL gene and transcript annotations including non-coding RNAs were used. Expression level of features, i.e. genes and non-coding RNAs, were quantified using FeatureCounts as raw read counts (12).

Differential expression statistics (fold-change and p-value) were computed using edgeR on raw expression counts obtained from quantification (13, 14). Raw expression counts were normalized within edgeR using TMM normalization. Nominal p-values were adjusted for multiple testing using the false discovery rate (FDR) correction of Benjamini and Hochberg (15). Significant genes were determined based on fold-changes lower than 0.5 and higher than 2.0, FDR threshold of 10% (q-value<0.1) in the multi-group comparison. Processed data on gene expression levels are provided in **Supplementary Table 3**.

Regulatory pathway analysis was performed in Gene Set Enrichment Analysis (GSEA) software (University of California San Diego and Broad Institute, USA) (16, 17) using the list of genes displaying significant differential expression in KG-1 cells treated with MK-2206. Input data contained Log_2_-fold-change values. GSEAPreranked algorithm was used to analyze data using Pathway Interaction Database collection of gene signatures (18) with number of permutations set to 1 000. Pathways with FDR<0.05 were considered significantly enriched.

### Annexin V-Based Detection of Apoptotic Cells

Cells were treated with drugs as described above in 60 mm cell culture dishes (Thermo Fisher Scientific). After treatment the cells were harvested by centrifugation, washed twice with ice-cold PBS, and 500 000 cells were resuspended in 100 μL of Annexin V Binding Buffer (BD Biosciences, San Jose, CA, USA). Cells were stained by incubating with 2.5 μL of APC-conjugated Annexin V recombinant protein (Thermo Fisher Scientific) for 15 minutes in the dark, pelleted by centrifugation at 200 g for 5 minutes, and resuspended in 300 μL of Annexin V Binding Buffer containing 0.1 μg/mL DAPI (R&D Systems). Samples were analyzed using CytoFLEX flow cytometer and CytExpert software (Beckman Coulter, Brea, CA, USA).

### Statistical Analysis

At least three independent biological replicates were used for all quantitative experiments describing cell treatment with drugs, excluding RNA-seq, where two biological replicates were used, and confirmatory Annexin V assay, where only one experiment was performed. RT-qPCR experiments were performed with two technical replicates for each biological replicate, gene expression levels were considered to be log-normally distributed (19) and differences between sample groups were evaluated using two-tailed Student’s t-test with Welch’s correction for unequal variances after logarithmic transformation of data. Protein levels estimated *via* immunoblotting were compared using Mann-Whitney U test. Correlation analysis of transcriptomic changes in “FOXM1-KD” and “MK-2206” genesets was performed for Log_2_-transformed fold-change data using Spearman’s correlation coefficient. Statistical significance was accepted with p<0.05. Statistical analysis was performed in OriginPro 2016 software (OriginLab Corporation, Northampton, MA, USA). Plots were generated using GraphPad Prism 6 software (GraphPad Software, San Diego, CA, USA).

Clustering of RNA sequencing data and heatmap plot generation were performed in Morpheus software (https://software.broadinstitute.org/morpheus, Broad Institute, USA) using Euclidean metrics and Complete linkage settings for clustering.

## Results

We investigated the role of FOXM1-AKT interconnection in AML using FLT3-wild-type KG-1 and THP-1 human AML cell lines, as FLT3-ITD mutation can promote AKT activity regardless of FOXM1 status. Using pharmacological inhibitors of AKT signaling pathway and FOXM1 function and stable shRNA-mediated FOXM1 knockdown (KD), we probed both “AKT-to-FOXM1” and “FOXM1-to-AKT” segments of FOXM1-AKT regulatory loop. We next compared the effects of AKT and FOXM1 inactivation using full transcriptomic RNA-seq analysis.

**Figure 1:**
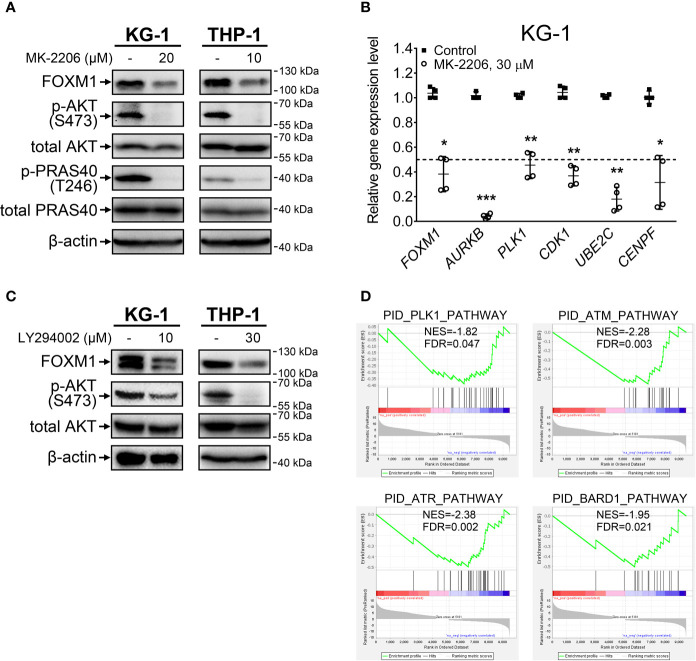
FOXM1 expression is regulated by the AKT pathway in AML. **(A)** Direct inhibition of AKT with MK-2206 results in FOXM1 suppression. Cells were treated with indicated concentrations of MK-2206 for 24 hours, total protein samples were purified immediately after treatment and analyzed *via* immunoblotting with indicated antibodies, β-actin was used as an internal loading control. **(B)** AKT inhibition causes downregulation of FOXM1 target genes expression. KG-1 cells were treated with 30 μM MK-2206 for 24 hours, total RNA was purified immediately after treatment and analyzed *via* RT-qPCR. Data are presented as individual data points and means ± S.D., statistical significance is evaluated for Log_2_-transformed data using two-tailed Student’s t-test with Welch’s correction (*p < 0.05, **p < 0.01, ***p < 0.001 for N = 4). **(C)** Inhibition of PI3K with LY294002 results in suppression of both AKT phosphorylation and FOXM1 levels. Cells were treated with indicated concentrations of LY294002 for 24 hours, total protein samples were purified immediately after treatment and analyzed *via* immunoblotting with indicated antibodies, β-actin was used as an internal loading control. **(D)** MK-2206 treatment of KG-1 cells suppresses genetic signatures of FOXM1 target PLK1 and DNA damage response regulators. KG-1 cells were treated with 30 μM MK-2206 for 24 hours, total RNA was purified immediately after treatment and analyzed *via* RNA-seq. NES, normalized expression score; FDR, false discovery rate.

Treatment with an allosteric pan-AKT inhibitor MK-2206 resulted in prominent inhibition of activating AKT phosphorylation at S473 site in both KG-1 and THP-1 cell lines (**Figure 1A**). We additionally confirmed that loss of AKT S473 phosphorylation correctly reflects the suppression of AKT pathway activity by demonstrating a decrease in T246 phosphorylation of direct AKT target protein PRAS40. In agreement with effects reported for solid cancers (8), AKT inhibition decreased FOXM1 protein levels in both examined cell lines (**Figure 1A**) and resulted in correspondent downregulation of established FOXM1 target genes (*AURKB*, *PLK1*, *CDK1*, *UBE2C*, *CENPF*) (**Figure 1B**).

To exclude the possibility that MK-2206 affects FOXM1 expression through a non-specific AKT-independent mechanism, we additionally treated cells with LY294002, a selective inhibitor of AKT upstream activator PI3K, and observed similar effects on phosphorylated AKT and FOXM1 protein levels (**Figure 1C**). These results for the first time experimentally confirm that activity of PI3K-AKT pathway is crucial for the maintenance of high FOXM1 expression levels in AML cells.

We next investigated the overall impact of AKT inhibition on gene expression patterns in AML *via* RNA-seq analysis of protein-coding transcriptome in KG-1 cells treated with MK-2206. We detected significant (at least 2-fold change, FDR<0.1) downregulation of 4 299 genes and upregulation of 5 181 genes (**Supplementary Table 3**). GSEA analysis performed using the list of significantly affected genes indicated suppression of PLK1 signature, providing additional evidence of FOXM1 suppression (**Figure 1D**). Moreover, we observed suppression of ATM, ATR, and BARD1 pathways involved in DNA damage response (DDR). Regulation of DDR *via* AKT pathway is well-established (20) and it can be partially facilitated through FOXM1 regulating the ATM-ATR signaling (21). The involvement of FOXM1 into DDR regulation emphasizes the importance of FOXM1-AKT loop in promoting AML cell survival and chemoresistance. Loss of the ATM-ATR signatures also suggest that AML cells with inhibited FOXM1/AKT activity may display higher sensitivity to anti-cancer therapy.

**Figure 2:**
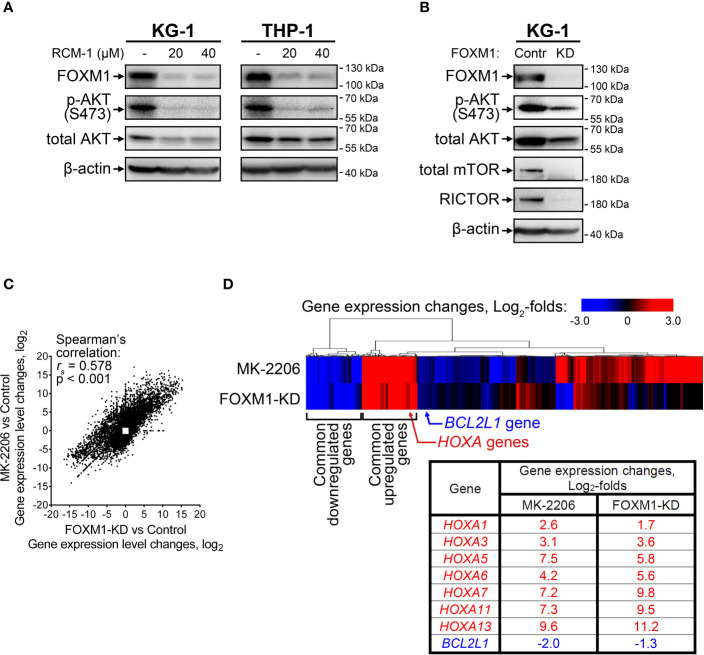
FOXM1 inactivation in AML cells decreases AKT activity and results in gene expression changes associated with venetoclax sensitivity. **(A)** FOXM1 inhibition with RCM-1 reduces phosphorylation and total protein levels of AKT. Cells were treated with indicated concentrations of RCM-1 for 72 hours, total protein samples were purified immediately after treatment and analyzed *via* immunoblotting with indicated antibodies, β-actin was used as an internal loading control. **(B)** Stable shRNA-mediated knockdown of FOXM1 in KG-1 cells suppresses the activity of mTORC2-AKT signaling axis. KG-1 cells were infected with lentiviral particles carrying a control or anti-FOXM1 shRNA-expressing vectors and selected in presence of puromycin. Total protein samples were purified after the selection and analyzed *via* immunoblotting with indicated antibodies, β-actin was used as an internal loading control. **(C)** Correlation between gene expression changes in KG-1 cells caused by MK-2206 treatment and FOXM1 knockdown effects. KG-1 cells were treated with 30 μM MK-2206 for 24 hours or transduced with FOXM1-KD or control lentiviral particles, RNA samples were purified from control, treated, and transduced cell cultures and subjected to RNA-seq, non-protein-coding genes were excluded from analysis. Data represent genes displaying significant (q<0.1) 2-fold or higher expression changes in at least one of the examined samples, average of two biological replicates for each condition. **(D)** Heatmap representation of MK-2206 treatment and FOXM1 knockdown effects on global gene expression patterns in KG-1 cells.

Since the positive regulation of FOXM1 by AKT pathway described in solid tumors was reproduced in AML cells, we hypothesized that reciprocal regulation of AKT by FOXM1 could also take place. To investigate this question, we treated KG-1 and THP-1 cells with a novel FOXM1 inhibitor RCM-1 (22, 23) and observed prominent decrease in both FOXM1 expression (5-fold or more) and AKT S473 phosphorylation (4-fold or more) levels. Moreover, we also detected slight (to 60-75% of control sample) suppression of total AKT protein levels in response to RCM-1 treatment (**Figure 2A** and **Supplementary Figure 1**). Published data on RCM-1 treatment effects in several different models suggest that it does not inhibit AKT phosphorylation in general (22). We therefore conclude that RCM-1-dependent inhibition of AKT pathway in AML cells may be mediated through FOXM1 suppression.

RCM-1 is a new drug and therefore may have unknown non-specific effects. To confirm that the observed RCM-1 treatment effects on AKT are mediated through FOXM1, we performed stable shRNA-mediated FOXM1 knockdown in KG-1 cells using lentiviral transduction approach. Similar to cells treated with RCM-1, FOXM1-deficient KG-1 cells displayed reduced levels of both phosphorylated and total AKT (**Figure 2B** and **Supplementary Figure 2**), confirming that FOXM1 positively regulates AKT on both protein and phosphorylation levels. To further explore possible regulatory mechanisms mediating FOXM1 effects on AKT, we evaluated levels of RICTOR, total mTOR and phosphorylated mTOR proteins in control and FOXM1-deficient KG-1 cells. Surprisingly, KG-1-FOXM1-KD cells displayed complete loss of both RICTOR and mTOR proteins (**Figure 2B** and **Supplementary Figures 2**, **3**), indicating that FOXM1 may be involved into regulation of their synthesis or degradation. Taken together with the data described above, our results indicate that AKT and FOXM1 in AML are organized into an autoregulatory loop that sustains elevated levels of FOXM1 and AKT activity.

To provide an additional confirmation of AKT-activating FOXM1 effect, we treated KG-1 cells with cytarabine (AraC), a drug commonly used in AML therapy. We previously demonstrated that AraC treatment increases FOXM1 level in KG-1 cells (4). As we expected, AraC-treated KG-1 cells also displayed increase in phospho-AKT levels. More importantly, addition of RCM-1 to AraC prevented both FOXM1 and phospho-AKT upregulation (**Supplementary Figure 4**).

We then compared gene expression changes induced in KG-1 cells by either AKT inhibition (“MK-2206” geneset) or stable FOXM1 knockdown (“FOXM1-KD” geneset) using RNA-seq. A total of 10 698 protein-coding genes were significantly affected in either one or both examined samples with 3 578 genes (33.4%) displaying co-directional changes in both samples (**Supplementary Table 3**). “MK-2206” and “FOXM1-KD” genesets exhibited prominent positive correlation (**Figure 2C**) with two clearly discernible groups of common up- and down-regulated genes identified by unsupervised hierarchical clustering (**Figure 2D**). This overlap of transcriptomic effects caused by inactivation of either AKT or FOXM1 confirms the idea that these two regulators share several common downstream effectors and strongly implies that FOXM1 and AKT are incorporated into a single regulatory network. We analyzed the clusters of overexpressed and repressed genes common to both “MK-2206” and “FOXM1-KD” genesets looking for genes potentially associated with AML drug sensitivity and detected increase in the expression of multiple genes belonging to *HOXA* family (*HOXA1*, *HOXA3*, *HOXA5*, *HOXA6*, *HOXA7*, *HOXA11*, *HOXA13*) (**Figure 2D**). The expression changes of the most abundant *HOXA* variants were additionally confirmed by RT-qPCR (**Supplementary Figure 5**). We also detected suppression of *BCL2L1* (encodes anti-apoptotic Bcl-xL protein), but the extent of this change was less than in case of *HOXA* genes (**Figure 2D**).

**Figure 3:**
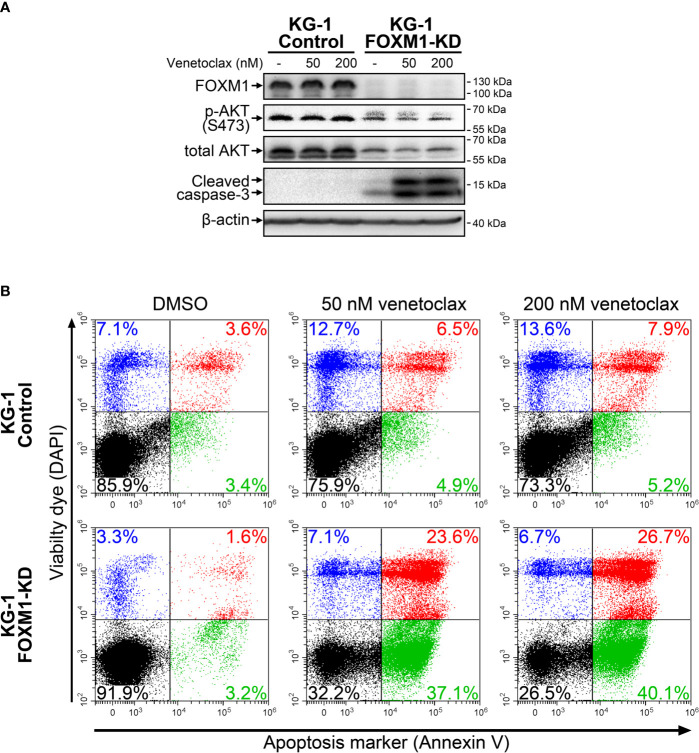
FOXM1 knockdown in KG-1 cells increases their sensitivity to venetoclax. KG-1-Control and KG-1-FOXM1-KD cells were treated with indicated concentrations of venetoclax for 24 hours. **(A)** Total protein samples were purified immediately after treatment and analyzed *via* immunoblotting with indicated antibodies. Apoptotic activity was evaluated based on caspase-3 cleavage, β-actin was used as an internal loading control. **(B)** Cells were harvested immediately after treatment and stained with Annexin V and DAPI. Flow cytometry-based Annexin V assay was performed to identify viable (DAPI^low^/Annexin V^low^, black), early apoptotic (DAPI^low^/Annexin V^high^, green), early necrotic (DAPI^high^/Annexin V^low^, blue) and dead (DAPI^high^/Annexin V^high^, red) cells.

High *HOXA* expression in AML was recently reported to be associated with increased sensitivity to venetoclax treatment, while increased *BCL2L1* level confer resistance to this drug (24, 25). We therefore evaluated its cytotoxic effects in control and FOXM1-deficient KG-1 cells using immunoblotting and Annexin V assay. KG-1-Control cells were resistant to venetoclax (up to 200 nM), while KG-1-FOXM1-KD cells demonstrated prominent activation of caspase-3 after 24 hours of treatment (**Figure 3A**). Immunoblotting results were verified using Annexin V assay that confirmed high percentage (60+%) of cells undergoing early and late apoptosis in FOXM1-deficient cells treated with venetoclax, but not in KG-1-Control cells (**Figure 3B**). Based on these results, we conclude that the positive regulatory loop existing between FOXM1 and AKT pathway is involved in the maintenance of venetoclax resistance in AML.

## Discussion

Present study was focused on the potential role of reciprocal regulation of FOXM1 and AKT in AML. Positive regulatory loop responsible for maintenance of high AKT and FOXM1 activity was described in multiple solid tumors but never in AML (1). We demonstrated for the first time that FOXM1-AKT loop exists in AML cells, and that the inactivation of either AKT or FOXM1 results in coordinated inhibition of its counterpart. Positive regulation of FOXM1 by AKT is in agreement with prior report of FOXM1 overexpression in AML being dependent on FLT3-ITD mutation that results in constitutive activation of AKT pathway (26). In contrast, a positive feedback link leading from FOXM1 back to AKT in AML is a completely new finding that reinforces FOXM1 as a promising therapeutic target.

While the exact regulatory pathways facilitating FOXM1 effects on AKT signaling are currently unknown, our surprising discovery of mTOR and RICTOR loss in FOXM1-deficient KG-1 cells may shed some light on this area. To our knowledge, this is the first known evidence of FOXM1 regulating mTOR-RICTOR-AKT axis by affecting total protein levels, since the activity of these proteins is usually controlled through phosphorylation. None of *MTOR*, *RICTOR*, or *AKT* genes were ever reported as FOXM1 targets; moreover, none of these genes display significant expression changes in our RNA-seq data (**Supplementary Table 3**). We therefore suppose that FOXM1 inactivation may result in either reduced translation or increased degradation of mTOR, RICTOR, and AKT proteins, but this hypothesis requires extensive additional studies.

AKT promotes FOXM1 expression *via* phosphorylating and inactivating FOXO3, another Forkhead box transcription factor that competes with FOXM1 for the same DNA sites. Phosphorylated FOXO3 protein is translocated to the cytoplasm and degraded, leaving its binding sites available for FOXM1 (8). One such site is situated in FOXM1 promoter and establishes self-activating loop involved in FOXM1 overexpression (27). In normal cells, FOXO3 re-accumulates in nucleus after AKT pathway inactivation, occupies FOXM1 promoter, and thus suppresses FOXM1 expression. However, the formation of FOXM1-AKT loop in AML cells likely results in constitutive hyperactivation of AKT pathway (even in absence of activating mutations) and constant inactivation of FOXO3, disabling this mechanism of FOXM1 overexpression prevention.

FOXM1 overexpression is known for its association with resistance to anti-cancer drugs, especially to DNA-damaging agents (21, 28). AML is not an exception, since nuclear FOXM1 promotes its resistance to AraC (5). Moreover, AKT pathway is also a regulator of DDR (20), and this fact is supported by our RNA-seq data for MK-2206-treated cells. Interestingly, FOXM1 itself can regulate the ATM-ATR signaling (21) and therefore is likely involved in facilitating AKT-dependent regulation of DDR. These complex connections between FOXM1, AKT and DDR emphasizes the importance of FOXM1-AKT loop in promoting AML cell survival and chemoresistance.

While FOXM1 role in general chemoresistance is well established, the sensitization of FOXM1-deficient cells to venetoclax, a specific Bcl-2-targeting agent, was unpredicted. Currently known mechanisms of venetoclax resistance development include loss of Bcl-2 (due to mutations or transcriptional repression) and overexpression of Bcl-xL and MCL-1 proteins (25). Our transcriptomic data indicate that KG-1 cells express moderate levels of *BCL2*, while no significant decrease in *MCL1* expression levels occurs upon MK-2206 treatment or FOXM1 knockdown (**Supplementary Table 3**). However, *BCL2L1* expression is decreased in KG-1 cells after FOXM1 or AKT inactivation, and this effect may be a potential mechanism for sensitization toward venetoclax. The information on possible FOXM1 and Bcl-xL connections is very scarce and is limited to two reports describing positive correlation between these proteins in thyroid cancer (29) and pancreatic β-cells (30). On the other hand, the involvement of AKT-Bcl-xL axis in venetoclax resistance in leukemia was reported before (31, 32), and AKT pathway inhibition may facilitate the suppression of *BCL2L1* in KG-1-FOXM1-KD cells. We therefore suggest that the whole FOXM1-AKT feedback loop is involved in AML venetoclax sensitivity regulation, while disruption of this loop at any point (AKT, FOXM1, or intermediate regulators) may help to overcome drug resistance. Elucidating this mechanism further has the potential to improve the therapeutic efficacy of Bcl-2 targeting strategies.

Mutations of *NPM1* are associated with a favorable outcome for AML patients. This effect could be partially explained by relocalization of FOXM1-NPM^mut^ complex to the cytoplasm, resulting in functional FOXM1 inactivation, disruption of FOXM1-AKT loop, and increased chemosensitivity (4, 5). AML cases harboring *NPM1* ^mut^ also display sensitivity to hypomethylating agents and venetoclax (33). *NPM1* mutations in AML are also associated with high expression of *HOX* genes (34) that has been reported as a marker of venetoclax sensitivity in primary AML samples (24). Importantly, our data demonstrate a very similar pattern of *HOXA* genes overexpression in KG-1 cells with FOXM1 deficiency or inhibited AKT (**Figure 2D**). Furthermore, FOXM1 knockdown in KG-1 cells sensitizes them to venetoclax (**Figure 3**). Thuswise, a very prominent association between FOXM1-AKT loop inactivation, *HOXA* genes expression, treatment sensitivity and *NPM1* mutations is reproduced in multiple AML model systems and sample sets, implying that these aspects are regulated by a common circuit. Our data suggest that strong *HOX* gene expression in *NPM1* ^mut^ AML cells (28) may be partly explained by inactivation of FOXM1-AKT loop (**Figure 2D**). We therefore propose that FOXM1-AKT positive regulatory loop acts as an important mechanism contributing to AML treatment resistance, and its disruption may be a universal way to induce drug sensitivity.

AKT pathway is an attractive target due to its involvement in multiple cancer-related processes. However, direct AKT inhibitors displayed subpar efficacy in clinical trials, probably due to high toxicity exerted by complete inhibition of AKT signaling (35). We propose that FOXM1-AKT positive feedback is responsible for maintenance of elevated AKT activity in AML. The disruption of this regulatory circuit in cells that do not have other mechanisms of constitutive AKT activation (e.g. FLT3-ITD mutation) may return AKT pathway activity back to baseline levels. Such effect would potentially avoid the risk for high toxicity of complete AKT inactivation and would not affect non-malignant cells that typically do not display high FOXM1 levels, while still providing anti-cancer effects of the AKT activity suppression. Specific pharmacological inhibition of FOXM1 activity is still a challenging task, but further investigation of molecular mechanisms facilitating AKT pathway regulation by FOXM1 may provide new approaches to inhibition of FOXM1-AKT regulatory loop and overcoming AML treatment resistance.

## Data Availability Statement

The datasets presented in this study can be found in online repositories. The names of the repository/repositories and accession number(s) can be found below: GEO, GSE172189.

## Author Contributions

MC conceptualized experimental part of the study, designed and performed experiments, acquired, analyzed, and interpreted data, drafted and revised the manuscript. SB designed and performed the experiments, acquired, analyzed, and interpreted data, drafted and revised the manuscript. MH designed and performed the experiments, acquired and analyzed data, revised the manuscript. ZA performed RNA sequencing experiments, drafted the manuscript. IK conceptualized experimental part of the study, analyzed and interpreted data, revised the manuscript, provided funding, supervised the study. AG proposed original idea, conceptualized the study, interpreted data, drafted and revised the manuscript, provided funding, supervised the study. All authors contributed to the article and approved the submitted version.

## Funding

The study was supported by LRF grant awarded to IK and UIC Cancer Center 2020 grant awarded to ALG.

## Conflict of Interest

The authors declare that the research was conducted in the absence of any commercial or financial relationships that could be construed as a potential conflict of interest.

## Publisher’s Note

All claims expressed in this article are solely those of the authors and do not necessarily represent those of their affiliated organizations, or those of the publisher, the editors and the reviewers. Any product that may be evaluated in this article, or claim that may be made by its manufacturer, is not guaranteed or endorsed by the publisher.
