# Supplementary material for: FOXM1-AKT Positive Regulation Loop Provides Venetoclax Resistance in AML
Source: Front Oncol. 2021 Jul 26;11:696532. doi: 10.3389/fonc.2021.696532 (PMC8350342; doi:10.3389/fonc.2021.696532)
Supplement: Supplementary file 1 [file Table_1.docx]

Supplementary Material

# Supplementary Table 1

List of antibodies used for immunoblotting.

| Protein target | Antibody supplier | Antibody host and clone/ID | Antibody working dilution |
| --- | --- | --- | --- |
| FOXM1 | Cell Signaling Technology, USA | Rabbit monoclonal, D12D5 | 1:500 |
| FOXM1 | Santa Cruz Biotechnology, USA | Rabbit monoclonal, C-20 | 1:1 000 |
| Phospho-AKT (S473) | Cell Signaling Technology, USA | Rabbit monoclonal, D9E | 1:1 000 |
| Total AKT (pan) | Cell Signaling Technology, USA | Rabbit monoclonal, C67E7 | 1:1 000 |
| Phospho-PRAS40 (T246) | Cell Signaling Technology, USA | Rabbit monoclonal, C77D7 | 1:1 000 |
| Total PRAS40 | Cell Signaling Technology, USA | Rabbit monoclonal, D23C7 | 1:1 000 |
| RICTOR | Cell Signaling Technology, USA | Rabbit monoclonal, 53A2 | 1:1 000 |
| Phospho-mTOR (S2448) | Cell Signaling Technology, USA | Rabbit monoclonal, D9C2 | 1:1 000 |
| Total mTOR | Cell Signaling Technology, USA | Rabbit monoclonal, 7C10 | 1:1 000 |
| Cleaved caspase-3 | Cell Signaling Technology, USA | Rabbit monoclonal, 5A1E | 1:1 000 |
| β-actin | MilliporeSigma, USA | Mouse monoclonal, AC-15 | 1:20 000 |
| anti-rabbit IgG, HRP-conjugated | Jackson Immunoresearch, USA | Alpaca polyclonal, 611-035-215 | 1:10 000 |
| anti-mouse IgG, HRP-conjugated | Jackson Immunoresearch, USA | Donkey polyclonal, 715-035-150 | 1:10 000 |

# Supplementary Table 2

List of primers used in RT-qPCR analysis.

| Gene | Forward primer | Reverse primer |
| --- | --- | --- |
| *GAPDH* | CCATCTTCCAGGAGCGAG | CTTGAGGCTGTTGTCATACTTC |
| *FOXM1* | CGAAAGATGAGTTCTGATGGAC | TCCTCTCAGTGCTGTTGATG |
| *AURKB* | AAGGGAGAGCTGAAGATTGC | TGAGCAGTTTGGAGATGAGG |
| *PLK1* | ACGGCTTTTTCGAGGACAAC | TGGCAGCCAAGCACAATTTG |
| *CDK1* | GATCTACCATACCCATTGACTAAC | ATGGCTACCACTTGACCTG |
| *UBE2C* | CATTGATAGTCCCTTGAACACAC | CTGCTTTGAGTAGGTTTCTTGC |
| *CENPF* | CAGGCTTTGCTTTTGGACAC | GGGCACGTTCATTTTCCTTC |
| *HOXA1* | CCTCCCAAAACAGGGAAAG | GGAGAGATGGGCAAGAGAC |
| *HOXA7* | CCTGGATGCGGTCTTCAG | CTTCGTCCTTATGCTCTTTCTTC |
| *HOXA11* | GCCACACTGAGGACAAGG | AGACGCTGAAGAAGAACTCC |


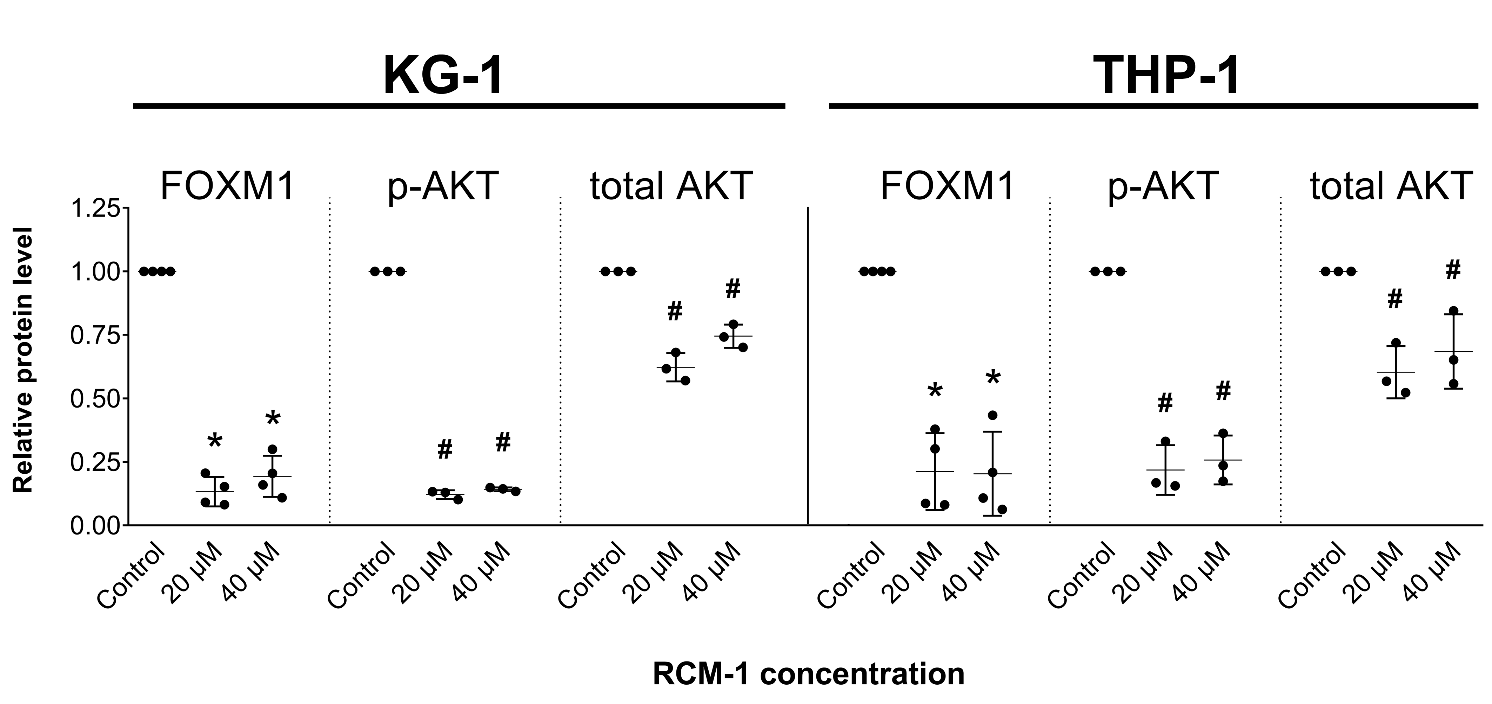


# Supplementary Figure 1. FOXM1 inhibition in AML cells decreases AKT phosphorylation and total protein levels. Cells were treated with indicated concentrations of RCM-1 for 72 hours, total protein samples were purified immediately after treatment and analyzed via immunoblotting with indicated antibodies, β-actin was used as an internal loading control (see Figure 2A). Band intensity in each independent experiment was estimated via densitometry and normalized to corresponding β-actin levels and Control sample. Data are presented as individual data points and means±S.D., statistical significance is evaluated using Mann-Whitney U test (*p=0.029 for N=4, ^#^p=0.1 for N=3).


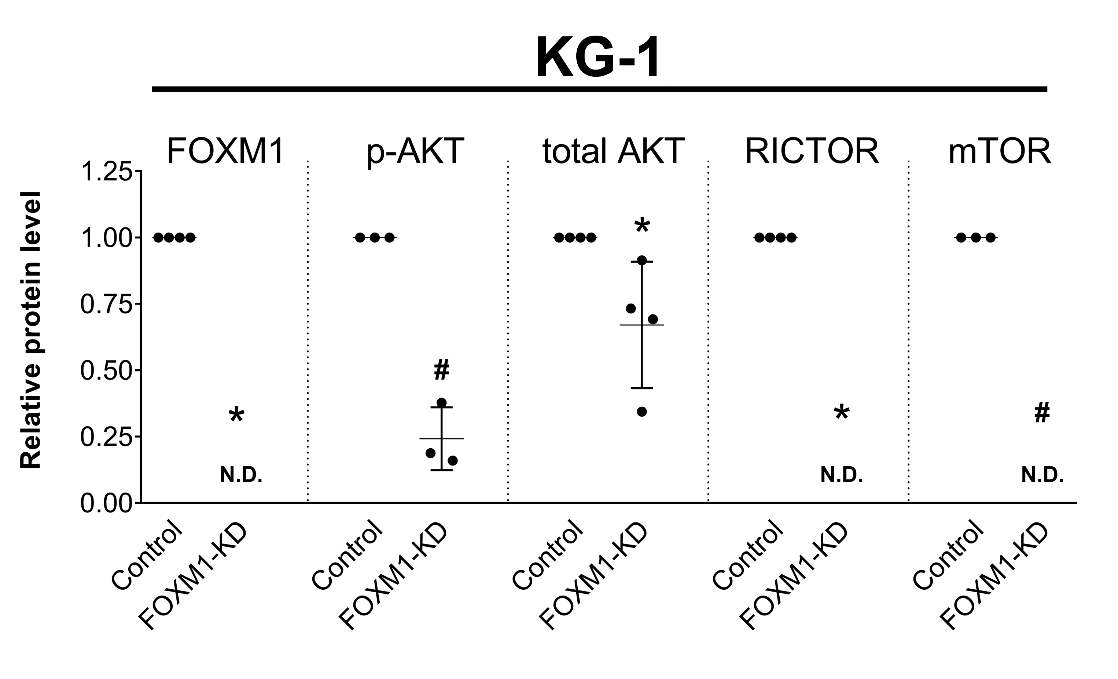


# Supplementary Figure 2. FOXM1 knockdown in KG-1 cells results in suppression of mTORC2-AKT signaling axis elements. KG-1 cells were infected with lentiviral particles carrying a control or anti-FOXM1 shRNA-expressing vectors and selected in presence of puromycin. Total protein samples were purified after the selection and analyzed via immunoblotting with indicated antibodies, β-actin was used as an internal loading control (see Figure 2B). Band intensity in each independent experiment was estimated via densitometry and normalized to corresponding β-actin levels and Control sample. Data are presented as individual data points and means±S.D., statistical significance is evaluated using Mann-Whitney U test (*p=0.029 for N=4, ^#^p=0.1 for N=3). N.D. – bands were not detected in any of the experiments.


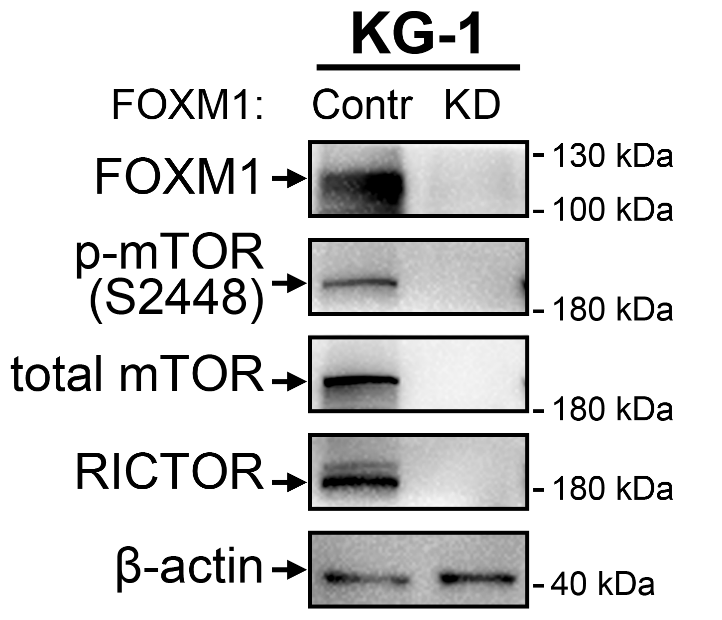


# Supplementary Figure 3. FOXM1 knockdown in KG-1 cells results in suppression of mTORC2 complex components. KG-1 cells were infected with lentiviral particles carrying a control or anti-FOXM1 shRNA-expressing vectors and selected in presence of puromycin. Total protein samples were purified after the selection and analyzed via immunoblotting with indicated antibodies, β-actin was used as an internal loading control.


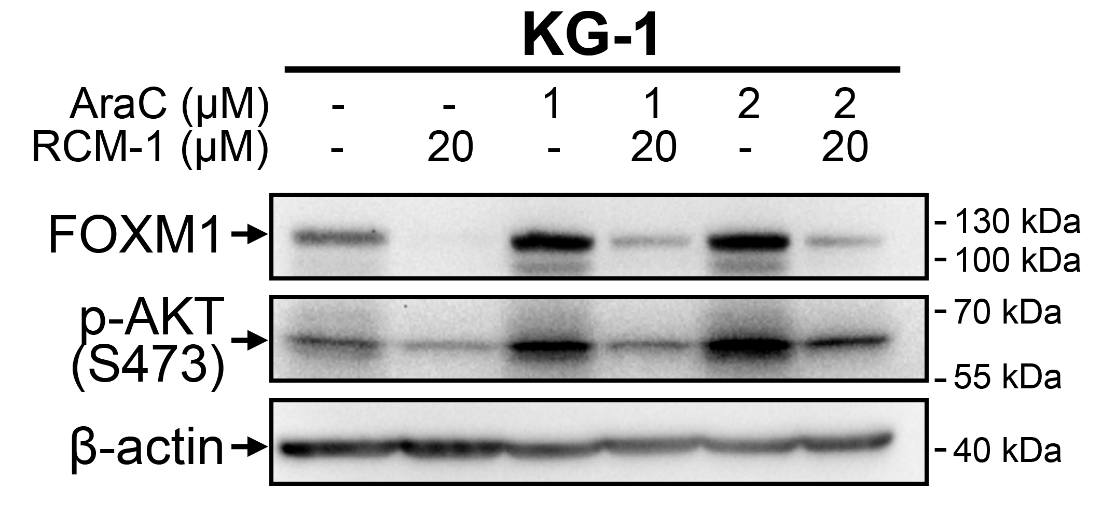


# Supplementary Figure 4. AraC-induced FOXM1 overexpression in KG-1 cells is associated with AKT activation. KG-1 cells were pre-treated with 20 μM of RCM-1 for 48 hours and then treated with indicated concentrations of RCM-1 and AraC alone or in combinations for 24 hours. Total protein samples were purified immediately after treatment and analyzed via immunoblotting with indicated antibodies, β-actin was used as an internal loading control.


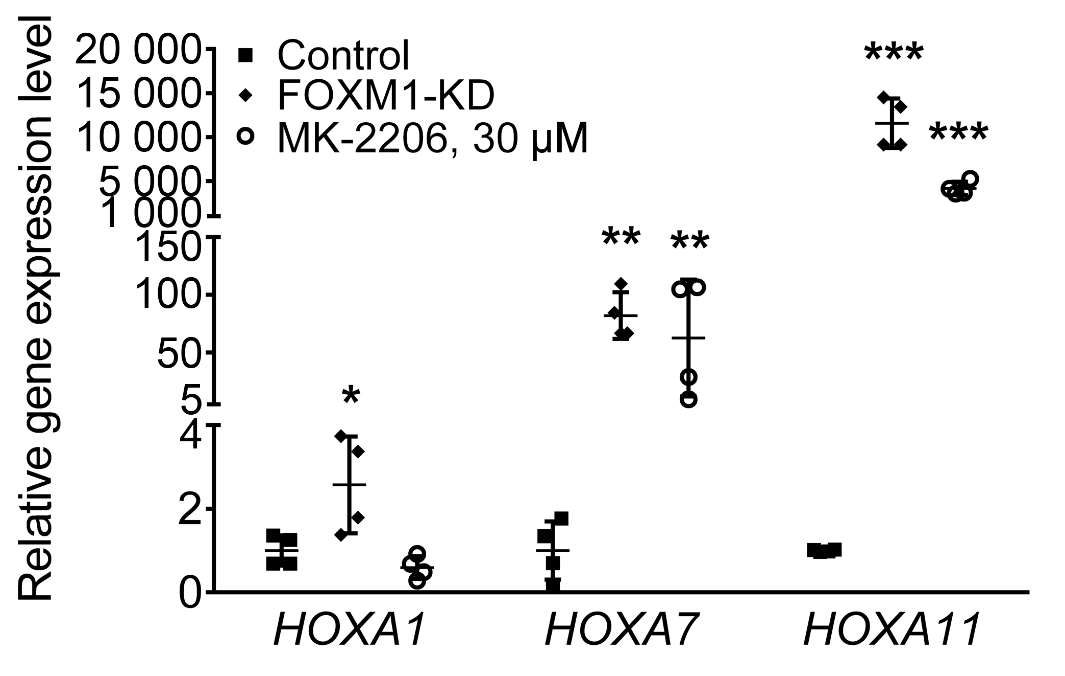


# Supplementary Figure 5. FOXM1 knockdown and AKT inhibition in KG-1 cells result in upregulation of multiple HOXA genes. KG-1 cells were treated with 30 μM MK-2206 for 24 hours or transduced with FOXM1-KD lentiviral particles, RNA samples were purified from control, treated, and transduced cell cultures and analyzed via RT-qPCR. Data are presented as individual data points and means±S.D., statistical significance is evaluated for Log_2_-transformed data using two-tailed Student’s t-test with Welch’s correction, (*p<0.05, **p<0.01, ***p<0.001 for N=4).
